# Exploring the effects of combined nostalgic activities and music therapy on Alzheimer's disease outcomes

**DOI:** 10.3389/fpsyg.2025.1526761

**Published:** 2025-01-30

**Authors:** Yunqiu Zhang, Yunqiong Wang, Qiao Liu, Jun Xiao, Ling Huang, Lan Zhou, Xuemei Liu

**Affiliations:** Sichuan Provincial Center for Mental Health, Sichuan Provincial People's Hospital, School of Medicine, University of Electronic Science and Technology of China, Chengdu, China

**Keywords:** Alzheimer's disease, nostalgic music therapy, cognitive function, negative emotions, sleep quality

## Abstract

**Objective:**

Exploring the effects of combination of nostalgic activity-based therapies, including music therapy on cognitive function, negative emotions, and sleep quality in patients with mild to moderate Alzheimer's disease.

**Methods:**

A total of 63 patients with mild to moderate Alzheimer's disease who were treated at the Sichuan Provincial Psychiatric Center of the People's Hospital of Sichuan Province from January to June 2023 were selected as the research subjects. They were randomly divided into a study group (*n* = 31) and a control group (*n* = 32) using a random number table method. The control group received routine treatment and nursing care, while the study group received nostalgic music therapy intervention on the basis of the control group. The Mini Mental State Examination (MMSE), Montreal Cognitive Assessment Scale (MOCA), Self Rating Anxiety and Depression Scale (SAS, SDS), and Pittsburgh Sleep Quality Index (PSQI) of the two groups were compared.

**Results:**

A total of 30 cases from each group completed the study. After 12 weeks of intervention, the MMSE and MOCA scores of both groups of patients increased, and the treatment group was higher than the control group (*P* < 0.05); SAS, SDS and PSQI scores decreased compared with those before intervention, and the treatment group was lower than the control group (*P* < 0.05).

**Conclusion:**

Nostalgic music therapy can improve cognitive function, alleviate negative emotions, and improve sleep quality in patients with mild to moderate Alzheimer's disease.

## 1 Introduction

Alzheimer's disease (AD) is an irreversible neurodegenerative disease characterized by acquired cognitive impairment. Its primary clinical manifestations include progressive intellectual decline, behavioral abnormalities, and deterioration in daily activities (Wang et al., 2024). Due to the aging population, AD has become a leading cause of disability and death among the elderly (Safiri et al., 2024). In China, the number of individuals over 60 years old diagnosed with AD has reached 9.83 million, with prevalence progressively increasing with age. As the disease advances, it affects basic bodily functions such as walking and swallowing, significantly reducing the quality of life. This condition not only reduces the quality of life for the elderly but also imposes a substantial burden on families and society (2017). While medications can enhance physical function and sleep quality in patients, they are often associated with side effects such as nausea, loss of appetite, diarrhea, vomiting, and weight loss (Jia et al., 2020). These limitations have driven the exploration of non-pharmacological interventions, which offer safer, low-cost alternatives to alleviate AD symptoms. Thus, non-pharmacological interventions—including exercise, social activities, music therapy, cognitive challenges, and a balanced diet—are recommended as preventive measures with fewer side effects (Mühlbauer et al., 2021; Langoni et al., 2019; Higuti et al., 2021).

Music therapy is a one such non-pharmacological intervention that has garnered attention due to its potential to maintain and improve cognitive function and social behavior in Alzheimer's disease patients (Cuddy et al., 2015; Li et al., 2015). Numerous studies have shown that music therapy can enhance various cognitive and psychological aspects, including attention, memory, orientation, depression, and anxiety (Shokri et al., 2023; Raglio et al., 2014; Sun et al., 2024). Non-pharmacological treatments have become essential in both preventing and alleviating symptoms of AD. Music therapy (Moreira et al., 2023) and reminiscence therapy (Bleibel et al., 2023), in particular, work by stimulating the brain to awaken individuals' positive memories, with meaningful music further enhancing the recall experience. These therapies have shown positive effects on patients' physical, psychological, and social wellbeing, although few studies have examined the impact of their combined intervention. Bayram (2024) found that activities and discussions centered around family or traditional cultural experiences are crucial in reminiscence therapy. Music associated with cultural themes, moreover, can evoke positive memories and enhance subjective wellbeing (Macleod et al., 2021). While music therapy has been extensively studied, the integration of cultural or nostalgic music with activities remains underexplored. Thus, incorporating music and activities rooted in traditional culture as core elements of reminiscence music therapy could represent a novel and effective strategy for symptom management in AD patients.

This study seeks to address these gaps by exploring the innovative integration of nostalgia-based music therapy with traditional cultural activities. The inclusion of cultural activities, such as those rooted in local customs and traditions, is hypothesized to create a richer, more meaningful context for therapy, thereby enhancing its efficacy. We hypothesize that the combination of nostalgia-based music therapy and culturally-themed activities, particularly those rooted in traditional Chinese solar terms, can produce a synergistic effect on the cognitive, emotional, and sleep outcomes of AD patients.

## 2 Materials and methods

### 2.1 Study design

**Figure 1:**
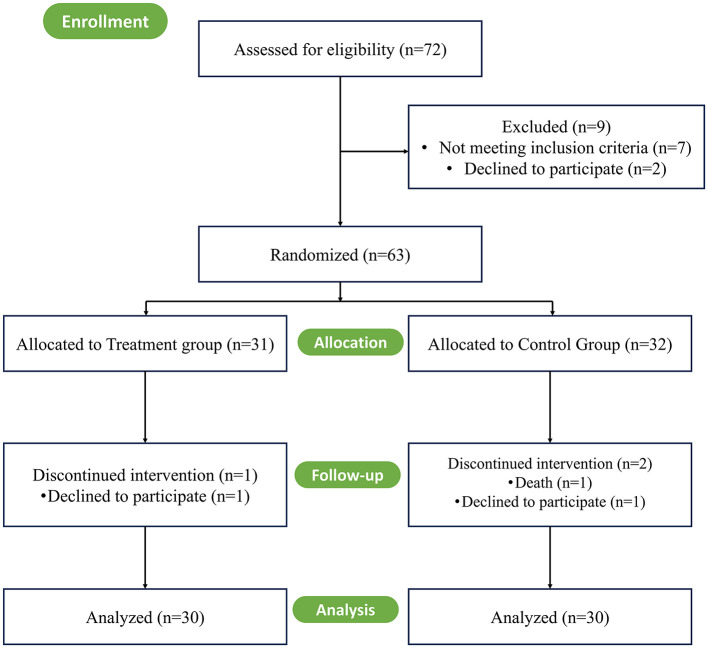
Schematic overview of study timeline (CONSORT flow diagram).

This study is a randomized clinical trial, patients with mild-to-moderate Alzheimer's disease (AD) who attended Sichuan Provincial People's Hospital or Sichuan Mental Health Center between January and June 2023 were selected as study subjects. Inclusion criteria included: (1) fulfillment of AD diagnostic criteria established by the NINCDS-ADRDA (Rio, 2018); (2) age ≥60 years, with ability to complete all neuropsychological assessments; and (3) a Mini-Mental State Examination (MMSE) score of 10–24. Exclusion criteria included: (1) significant auditory or visual impairment; (2) severe diseases of major organs (e.g., heart, liver, kidney) or other serious physical illnesses; and (3) history of organic or affective mental disorders. The cognitive functions were measured at the time of patient admission. Sample size was calculated using G-Power software with reference to previous studies (McKhann et al., 1984), assuming a 10% attrition rate, yielding a target of 30 cases per group. All participants in both the intervention and control groups continued their standard pharmacological treatments for Alzheimer's disease throughout the study. A total of 63 patients were enrolled, randomly allocated to the treatment group (*n* = 31) or control group (*n* = 32). A CONSORT flowchart of the present study is shown in Figure 1.

### 2.2 Training protocol

The control group received standard care, which included routine lifestyle care, health education, monitoring of patients' condition, administration of medications as prescribed, and participation in regular recreational activities. These activities included 30-min group sessions of Tai Chi or calisthenics each afternoon and singing or movie-watching sessions on Monday, Wednesday, and Friday afternoons. In addition to standard care, the treatment group received nostalgia-based music therapy. Since individuals from the same country often resonate with their traditional culture, this study was designed around Chinese traditional solar terms, using relevant music, customs, and activities as core elements. The intervention process was as follows: (1) Formation of a nostalgia music intervention team, consisting of one chief neurologist, two psychotherapists, three senior neurology nurses, and three nurse practitioners. The team discussed and revised the intervention plan, finalizing it following a pilot test within the hospital. The head nurse served as the team leader, providing pre-activity training to team members and clarifying their responsibilities. (2) Intervention methods: 12 significant Chinese solar terms, such as Beginning of Spring, Awakening of Insects, Pure Brightness, Grain in Ear, Summer Solstice, Great Heat, White Dew, Autumn Equinox, Descent of Frost, Beginning of Winter, Winter Solstice, and Great Cold, were chosen as activity themes. Each theme was accompanied by 10–15 images, a song, and interactive games related to the theme. Handcraft activities were included to improve participants' cognitive and coordination abilities, enhancing engagement and enjoyment. Participants were divided into three groups of 8–10 individuals each. (a) Each session began with a welcome song, followed by introductions among the leader and members (2 min); (b) Listening to and singing along with music related to the solar term (15 min); (c) Distributing images for participants to select those related to the theme and identify them correctly, followed by discussions of the theme and sharing positive memories or experiences associated with the solar term (15 min); (d) Engaging in traditional customs-themed games and handcraft activities, such as origami, paper cutting, creative drawing, and clay modeling, with participants free to choose based on their interests (25 min); (e) Concluding with a recap and singing a closing song (3 min). (3) Intervention duration: Each themed activity session lasted ~60 min, conducted once per week for a total of 12 weeks.

### 2.3 Outcome measures

Cognitive function, assessed using the Mini-Mental State Examination (MMSE) and the Montreal Cognitive Assessment Scale (MOCA). The MMSE provides a preliminary assessment of cognitive function for screening and follow-up, covering domains such as orientation, memory, calculation, attention, recall, naming, language, and visuospatial function (Faul et al., 2007). The MOCA includes spatial ability, naming, attention, sentence repetition, fluency, abstraction, delayed recall, and orientation, with a Cronbach's α coefficient of 0.933 and a correlation coefficient with the MMSE total score of 0.825, indicating high reliability and validity. Both scales have a maximum score of 30, with an MMSE score of ≥27 and a MOCA score of ≥26 indicating normal cognitive function.

Psychological status was assessed using the Self-Rating Anxiety Scale (SAS) and the Zung Self-Rating Depression Scale (SDS) before and after the intervention in both groups. The SAS and SDS each contain 20 items, with total scores multiplied by 1.25 and rounded to the nearest integer to obtain the standard score. Higher scores indicate more severe negative emotions (Folstein et al., 1975).

Sleep quality was assessed using the Pittsburgh Sleep Quality Index (PSQI) before and after the intervention in AD patients. This scale comprises 18 self-rated items grouped into seven components: sleep quality, sleep latency, sleep duration, sleep efficiency, sleep disturbances, use of sleep medication, and daytime dysfunction. Each component is rated on a scale from 0 to 3, with a total score ranging from 0 to 21. Higher scores indicate poorer sleep quality.

### 2.4 Ethical aspect

All procedures involving human participants were conducted in alignment with the Declaration of Helsinki. The Ethics Committee of Sichuan Provincial People's Hospital approved this study (approval number: 357[2021]).

### 2.5 Statistical analysis

Data analysis was performed using SPSS version 26.0 and Python 3, with data independently entered by two researchers. Variable normality was assessed via the Shapiro–Wilk test. Quantitative data are reported as mean ± standard deviation, while categorical data are presented as frequencies and percentages. Independent-sample *t*-tests, paired *t*-tests, and chi-square (χ^2^) tests were applied as appropriate, with statistical significance defined at α = 0.05.

## 3 Results

### 3.1 Baseline assessment

During the study, one case in the treatment group was lost due to health reasons, and two cases in the control group were lost due to disease progression and death, leaving 30 cases completed in each group. A comparison of baseline characteristics between the two groups showed no statistically significant differences (*P* > 0.05) (Table 1).

**Table 1:** Baseline characteristics of patients in the treatment and control groups before intervention.

	**Treatment group (*n* = 30)**	**Control group (*n* = 30)**	***P*-value**
**Gender**			0.605
Male	17 (56.7%)	14 (46.7%)	
Female	13 (43.3%)	16 (53.3%)	
Age (years)	65.0 [61.2 ;67.0]	64.5 [62.0; 67.8]	0.779
Duration of AD (years)	4.00 [3.00; 4.75]	4.00 [3.25; 5.00]	0.200
**Education level**			0.779
Primary school	5 (16.7%)	4 (13.3%)	
Junior high school	22 (73.3%)	20 (66.7%)	
Senior high school	3 (10.0%)	5 (16.7%)	
University	0 (0.00%)	1 (3.33%)	

### 3.2 Cognitive and emotional performance

**Figure 2:**
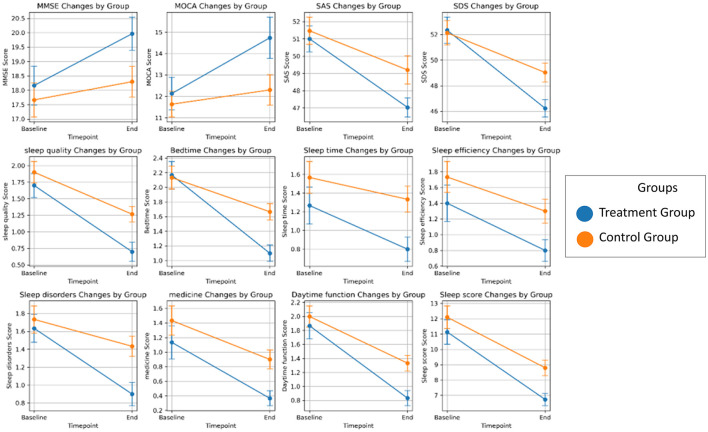
The scores of the cognitive, physical and sleep quality tests following the treatment.

There was no significant difference between groups at the MMSE and MOCA test at baseline (*p* > 0.05). As shown in Figure 2, both groups demonstrated an improvement in MMSE and MOCA scores from baseline after the intervention. Following the intervention, MMSE and MOCA scores in the treatment group were significantly higher than those in the control group (*P* < 0.05), as shown in Table 2. There were no significant differences in baseline SAS and SDS scores between the two groups (*P* > 0.05). As depicted in Figure 2, both groups showed a reduction in SAS and SDS scores following the intervention. The changes in SAS and SDS scores were significantly greater in the treatment group than in the control group (*P* < 0.05) (Table 3).

**Table 2:** Comparison of MMSE and MoCA scores in patients before and after intervention between treatment and control groups.

		**Treatment group (*n* = 30)**	**Control group (*n* = 30)**	***t***	***P*-value**
MMSE	Basal Mean (SD)	18.17 ± 3.69	17.67 ± 3.25	0.556	0.580
	Final Mean (SD)	19.97 ± 3.15^*^	18.30 ± 2.91^*^	2.129	0.038
MOCA	Basal Mean (SD)	12.13 ± 4.17	11.63 ± 3.27	0.517	0.607
	Final Mean (SD)	14.73 ± 5.27^*^	12.30 ± 3.88^*^	2.036	0.046

The indexes were compared with those before intervention. ^*^P < 0.05.

**Table 3:** Comparison of SAS and SDS scores in patients before and after intervention between treatment and control groups.

		**Treatment group (*n* = 30)**	**Control group (*n* = 30)**	***t***	***P*-value**
SAS	Basal Mean (SD)	51.00 ± 4.15	51.47 ± 4.29	−0.428	0.670
	Final Mean (SD)	47.03 ± 3.05^*^	49.20 ± 4.5^*^	−2.180	0.033
SDS	Basal Mean (SD)	52.33 ± 5.60	52.13 ± 5.18	0.144	0.886
	Final Mean (SD)	46.23 ± 3.72^*^	49.03 ± 4.06^*^	−2.787	0.007

The indexes were compared with those before intervention. ^*^P < 0.05.

### 3.3 Sleep quality

No significant differences were observed in baseline PSQI scores between the two groups. As shown in Figure 2, both groups exhibited a downward trend in the sleep quality, sleep onset time, total sleep time, sleep efficiency, sleep disorders, sleep aids, daytime dysfunction, and total score. Post-intervention, both the control and experimental groups showed statistically significant improvements in PSQI-related sleep indices. The experimental group showed significantly greater improvements in the total score and individual indicators compared to the control group, with statistical significance (Table 4). Overall, after 12 weeks of the training program, the experimental group exhibited significant improvements in all sleep performance indicators compared to the control group.

**Table 4:** Comparison of PSQI scores in patients before and after intervention between treatment and control groups.

	**Group**	**Treatment group (*n* = 30)**	**Control group (*n* = 30)**	***t***	***P*-value**
Sleep quality	Basal Mean (SD)	1.70 ± 0.99	1.90 ± 0.89	−0.428	0.670
	Final Mean (SD)	0.70 ± 0.79^*^	1.27 ± 0.64^*^	−2.180	0.033
Sleep onset time	Basal Mean (SD)	2.17 ± 1.02	2.13 ± 0.86	0.144	0.886
	Final Mean (SD)	1.10 ± 0.61^*^	1.67 ± 0.61^*^	−2.787	0.007
Total sleep time	Basal Mean (SD)	1.27 ± 1.08	1.57 ± 0.94	−1.150	0.255
	Final Mean (SD)	0.80 ± 0.71^*^	1.33 ± 0.76^*^	−2.804	0.007
Sleep efficiency	Basal Mean (SD)	1.40 ± 1.28	1.73 ± 1.08	−1.092	0.279
	Final Mean (SD)	0.80 ± 0.76^*^	1.30 ± 0.84^*^	0.279	0.019
Sleep disorders	Basal Mean (SD)	1.63 ± 0.85	1.73 ± 0.83	−0.462	0.646
	Final Mean (SD)	0.90 ± 0.71^*^	1.43 ± 0.63^*^	−3.081	0.003
Sleep aids	Basal Mean (SD)	1.13 ± 1.25	1.43 ± 1.10	−0.984	0.329
	Final Mean (SD)	0.37 ± 0.56^*^	0.90 ± 0.71^*^	−3.234	0.002
Daytime dysfunction	Basal Mean (SD)	1.87 ± 1.01	2.00 ± 0.83	−0.559	0.578
	Final Mean (SD)	0.83 ± 0.59^*^	1.33 ± 0.61^*^	−3.231	0.002
Total score	Basal Mean (SD)	11.13 ± 4.38	12.10 ± 4.05	−0.887	0.379
	Final Mean (SD)	6.73 ± 2.21^*^	8.80 ± 2.82^*^	−3.158	0.003

The indexes were compared with those before intervention. ^*^P < 0.05.

## 4 Discussion

The primary aim of this study is to assess the impact of combined nostalgic music therapy on cognitive function, negative emotions, and sleep quality in patients with mild to moderate Alzheimer's disease (AD). The results indicate that combined nostalgic music therapy resulted in significant improvements in cognitive function, negative emotions, and sleep quality compared to the control group. The findings suggest that incorporating combined nostalgic music and other cognitive stimuli into routine treatment and care may enhance neural activation and arousal, leading to improved psychological and physical outcomes and additional benefits.

Due to the aging population, AD has become a leading cause of disability and death among the elderly (Dunstan et al., 2017). In addition to the gradual decline in cognitive function, AD is often accompanied by emotional changes, behavioral abnormalities, negative emotions such as anxiety and depression, as well as sleep disorders and hallucinations (Jacobsen et al., 2015). The condition typically progresses over time, and most patients eventually lose their ability to live independently (García-Navarro et al., 2022). Numerous studies have shown that systematic, comprehensive, and regular non-pharmacological treatments can improve brain structure and functional plasticity in AD patients, alleviate clinical symptoms, delay disease progression, enhance social participation, and improve patients' quality of life (Jiménez-Palomares et al., 2024; Rajendran and Krishnan, 2024; Ting et al., 2024). For instance, Shokri et al. investigated the effects of remote music and exercise training on cognitive and physical functions in AD patients, reporting significant improvements in MMSE scores and physical performance measures in the group receiving combined music and exercise training compared to exercise-only and control groups (Shokri et al., 2023). Their findings underscore the importance of multi-modal interventions in addressing the multifaceted challenges of AD. Nostalgic music therapy utilizes traditional festivals, specific music, and past life experiences to stimulate the brain and awaken pleasant and meaningful memories related to past events, thus enhancing the recollection experience (Ismail et al., 2018). Research has shown that musical stimulation activates specific brain regions, such as the hypothalamus and hippocampus, and regulates cognitive and emotional functions (Plourde-Kelly et al., 2021). Chéour et al. (2023) conducted a randomized controlled trial to evaluate the effects of MT and physical rehabilitation (PR), either individually or in combination, on cognitive and motor functions in elderly Tunisian patients with mild AD. Their findings revealed that combined MT and PR interventions yielded the greatest improvements in cognitive (MMSE, ADAS-Cog) and motor (step length, walking speed, and BBS) functions, with MT showing a stronger influence on cognition and PR on motor abilities. Similar to these studies, our results show a positive impact of nostalgic music therapy on cognitive functions, as evidenced by significant improvements in the MMSE and MOCA scores. While Chéour et al. highlighted the synergistic effects of MT and PR, our study delves deeper into the cognitive and emotional impacts of nostalgic music therapy, specifically designed to evoke personal and cultural memories. Additionally, by helping patients recall the details of past experiences, it promotes neurogenesis and cell repair, thus enhancing cognitive function (Raglio et al., 2014). Our study expands on previous research by combining nostalgic music with other cognitive stimuli, demonstrating that such an integrative approach may enhance the therapeutic effects.

The prevalence of anxiety and depression in AD patients exceeds 60%, with over 50% of patients experiencing both anxiety and depression simultaneously. These emotional disturbances have a negative impact on cognitive function and overall health outcomes in AD patients (Pagonabarraga et al., 2023). Research has shown that music listening and recall can activate the brain's limbic and adjacent regions, while also promoting the release of biochemical mediators such as endorphins and dopamine, which induce a state of relaxation in patients (Saragih et al., 2022). In this study, nostalgic music therapy—designed around China's traditional solar terms—was shown to alleviate symptoms of anxiety and depression, which is consistent with findings from previous studies on music therapy The incorporation of personal experiences, such as events tied to the solar terms, and the use of photos or musical cues, facilitated the recall of positive memories and created a joyful atmosphere, thus improving emotional wellbeing. This highlights the importance of fostering positive memories and creating an emotionally supportive environment as key factors in alleviating negative emotions during the treatment and rehabilitation of AD patients. These results are consistent with previous research that suggests music and reminiscence therapies, particularly those focused on cultural experiences, help evoke positive memories and improve mood.

Sleep disturbances are a common clinical symptom of AD and also serve as a contributing and exacerbating factor. Approximately 50% of AD patients experience sleep disorders, which can result in complications such as immune dysfunction and neurotransmitter imbalances. These issues lead to progressive declines in patients' behavioral abilities, cognitive, and social functions, thereby increasing both the mental and physical burdens on caregivers (Lacerda et al., 2024). Therefore, enhancing sleep care for AD patients is critical for improving their health outcomes and reducing the burden on families and society. Studies indicate that active music therapy can effectively harmonize physiological, psychological, and emotional states, thus contributing to improved sleep quality (Keskin Töre and Yagmur, 2023; Lin et al., 2024; Lund et al., 2023; Mu et al., 2022). Results from this study indicate that patients receiving nostalgic music therapy had significantly lower PSQI scores across various domains and overall compared to the control group. This effect may be attributed to the therapy's use of traditional seasonal themes to create a comforting atmosphere, guiding patients in recalling pleasant memories from the past. This process, enhanced through music and interactive games, helped alleviate anxiety, thereby mitigating the negative impact of these emotions on sleep quality. Additionally, the interaction between musical sound waves and the brain can increase neuronal excitability, aiding in the regulation of circadian rhythms and ultimately supporting better sleep quality (Zaatar et al., 2024; Chen et al., 2023). Caregivers and therapists have observed that nostalgic music therapy brings positive experiences for patients across different sessions, stemming from memories evoked by music, past experiences, and connections with others. These observations should be interpreted with caution, and future studies should incorporate structured evaluations to validate these claims. This study was designed around the traditional Chinese solar terms, incorporating related music, customs, and activities as core elements for intervention in AD patients. In summary, the application of nostalgic music combination therapy effectively improves cognitive function in patients with mild to moderate AD, alleviates anxiety and depression, and enhances sleep quality.

However, this study has several limitations. These include a small sample size, the absence of long-term effect validation, and the potential confounding effect of continued pharmacological treatments, which cannot be entirely ruled out in the observed outcomes. Additionally, the short duration of the intervention (60 min per session) may limit the sustainability of the observed effects. The therapy duration was based on practicality and patient comfort, but longer sessions or more frequent interventions may yield stronger and more lasting outcomes. Furthermore, baseline characteristics, such as disease severity, comorbidities, and demographic factors, were not fully controlled for, which could introduce confounding variables. Moreover, the intervention was rooted in Chinese cultural practices, specifically the use of traditional solar terms and culturally significant music, which may limit its applicability in non-Chinese populations. Future studies should explore the effectiveness of culturally adapted versions of the therapy in diverse populations to assess their universal effectiveness. Another important limitation is the lack of long-term follow-up data to evaluate the sustainability of the effects of nostalgic music therapy. Future research should consider extending the duration of the intervention and conducting longitudinal studies to determine the lasting impact on cognitive function, emotional wellbeing, and sleep quality.

Future studies should explore several avenues to build upon the current findings. First, it is important to evaluate the differential impacts of live vs. recorded music to determine which format is more effective in enhancing cognitive function, emotional wellbeing, and sleep quality in AD patients. Additionally, research should focus on the long-term effects of nostalgic music therapy and whether its benefits are sustained over time. Finally, as our study utilized a culturally specific intervention, it would be valuable to investigate the applicability of similar therapies in non-Chinese populations to assess their universal effectiveness.

Despite these limitations, the study provides valuable insights into the potential role of nostalgic music therapy in improving outcomes for AD patients. The findings contribute to the broader understanding of AD care by demonstrating the potential of non-pharmacological interventions, particularly music therapy, in enhancing cognitive, emotional, and physical wellbeing. Our results suggest that nostalgic music therapy may be an effective, low-cost, and non-invasive addition to routine care plans for AD patients. Given its simplicity and accessibility, this intervention could be widely implemented in clinical and home settings, offering a complementary approach to traditional pharmacological treatments.

## 5 Conclusion

In conclusion, our findings suggest that integrating nostalgic music with routine daily care as an appropriate non-pharmacological intervention can improve cognitive function, alleviate negative emotions, and enhance sleep quality in Alzheimer's disease (AD) patients, while also reducing the societal burden of the disease. This approach not only offers potential benefits for patient wellbeing but also provides broader implications for Alzheimer's care by highlighting the value of culturally resonant, individualized therapeutic strategies within healthcare practices. Based on these results, we recommend that neurologists consider incorporating nostalgic music therapy alongside pharmacological treatments as part of the comprehensive and patient-centered approach to managing AD. However, our study is subject to certain limitations, including the small sample size, relatively short intervention duration, and its cultural specificity, which may limit the generalizability of the findings. Future research should focus on validating these outcomes in larger and more diverse populations, examining the long-term effects of combined nostalgic music therapy on cognitive and emotional health in AD patients.

## Data Availability

The original contributions presented in the study are included in the article/supplementary material, further inquiries can be directed to the corresponding author.
